# Data on quantum dot cellular automata based flip flops for designing serial-in-serial-out shift register

**DOI:** 10.1016/j.dib.2023.110019

**Published:** 2023-12-27

**Authors:** Birinderjit Singh Kalyan, Balwinder Singh, Rekha Devi

**Affiliations:** a I K Gujral Punjab Technical University, Jalandhar, Punjab, India; b Centre for Development of Advanced Computing (C-DAC), Mohali Punjab, India; c Chandigarh University, Mohali, Punjab, India

**Keywords:** QCA, D flip flop (D-FF), JK flip flop (JK-FF), QCA designer tool, Serial in Serial out Shift (SISO) register

## Abstract

There has been remarkable research carried out on Nano-electronics where Quantum dot Cellular automata emerge as the forthcoming paradigm in computing. The QCA-based circuits are used in the computational Nano hardware to present computations at ultra-high speed. A systematic approach has been utilized to design the Serial in Serial out Shift (SISO) Register using JK flip flop (JK-FF) and D flip flop (D-FF). These flip flops were initially designed with lower complexity which is the dominant factor for designing any complex sequential circuit. The QCA based designs have been validated and subjected to simulation using the QCA Designer tool ver. 2.0.3.

Specification Table

**Table:** 

Subject	Electronics
Specific subject area	Nanotechnology QCA Circuit Design
Data format	Analyzed
Type of data	Table, Figure
Data collection	The QCA Designer tool is used for designing and simulating QCA-based flip-flop circuits and serial-in/serial-out circuits based on the flip-flop design. Five-input majority gates were utilized to optimize the design, which was then implemented in the software with different clock cycles. D flip-flops and JK flip-flops were designed with an optimal cell count. Using these proposed optimal designs, serial-in-serial-out QCA-based circuits were designed. The cell count is then calculated and compared with previously reported designs. Furthermore, the quantum cost of the circuits was calculated based on the latency and complexity of the circuit.
Data source location	Chandigarh University, Mohali, Punjab, India I K Gujral Punjab Technical University, Jalandhar, Punjab, India
Data accessibility	Repository name: Mendeley data Data identification number: 10.17632/vk7t6f43hk.3 Direct URL to data: https://data.mendeley.com/datasets/vk7t6f43hk/3
Related research article	Kalyan, B. S., & Singh, B. (2020). Performance analysis of quantum dot cellular automata (QCA) based linear feedback shift register (LFSR). International Journal of Computing and Digital Systems, 9(03). DOI: http://dx.doi.org/10.12785/ijcds/090318

## 1 Value of the Data


 

• The D flip-flop and JK flip-flop QCA-based designs have been optimized to lower complexity by using the QCA Designer tool, employing majority gates. This has led to a reduction in the cell count of these circuits, consequently lowering their complexity during fabrication.
• The researcher involved in the development of quantum circuits will design complex sequential circuits using these basic flip-flops, such as shift registers, linear feedback shift registers, and counters.
• The researcher will reproduce and simulate the data using the QCA Designer tool. Complex sequential circuits can be optimized to reduce fabrication costs.
• Quantum cost is a significant factor in designing optimized quantum circuits.

## 2 Data Description

**Fig. 1:**
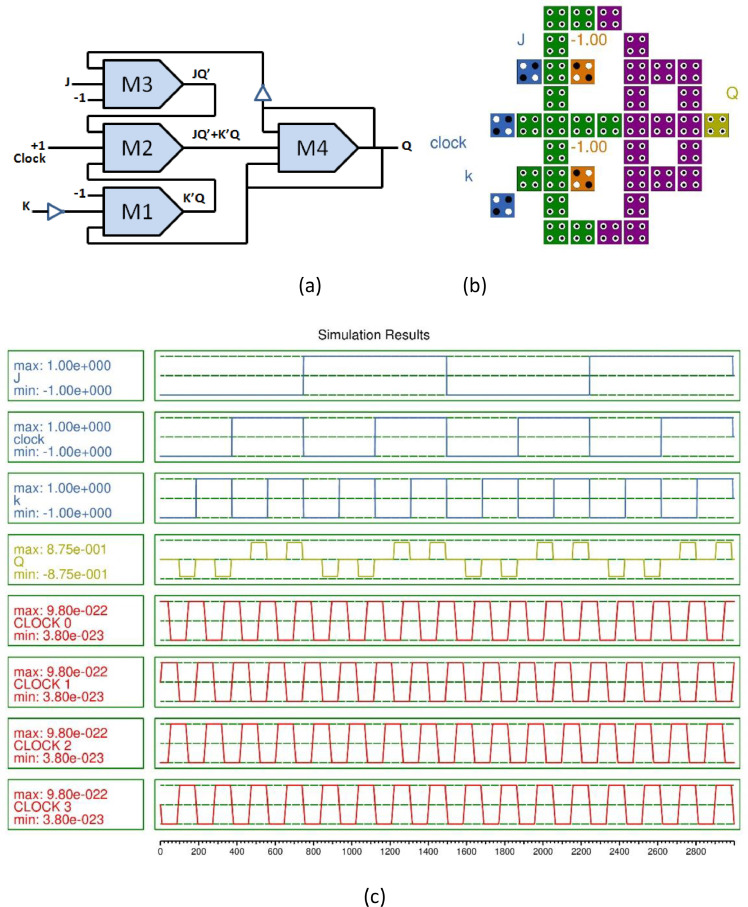
(a) Majority gate based JK-FF (b) QCA layout (c) Simulation results.

**Fig. 2:**
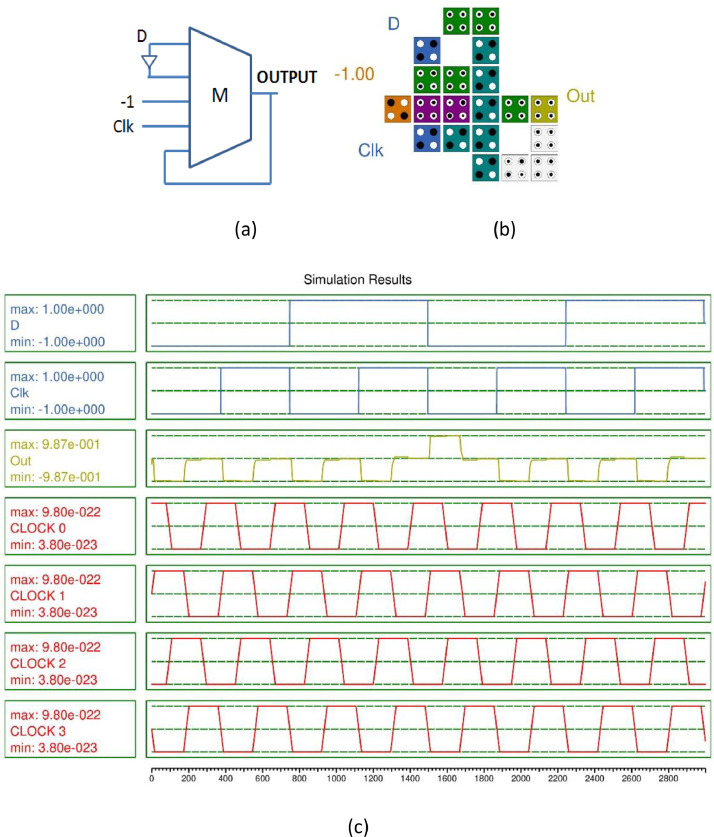
(a) Majority gate based D-FF (b) QCA layout (c) Simulation results.

**Fig. 3:**
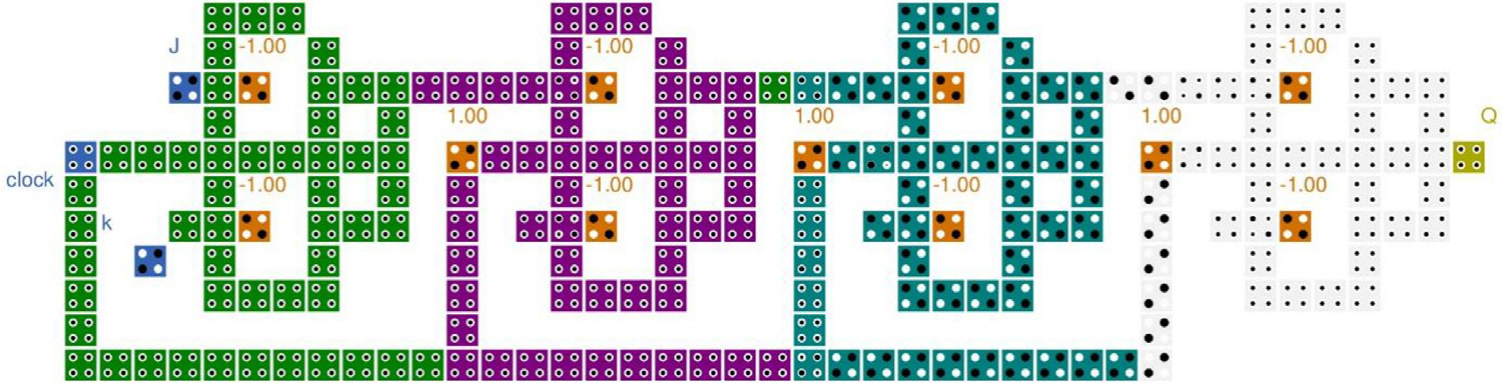
QCA based Shift register using JK-FF.

**Fig 4:**
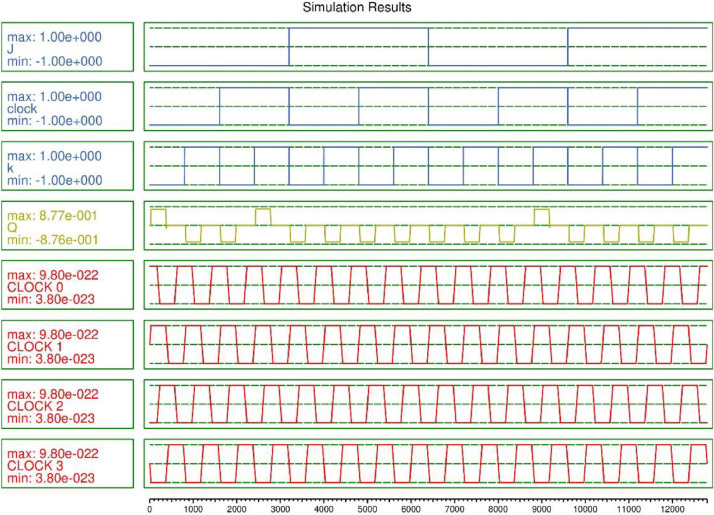
Simulation outcome of JK-FF based SISO Register.

**Fig. 5:**
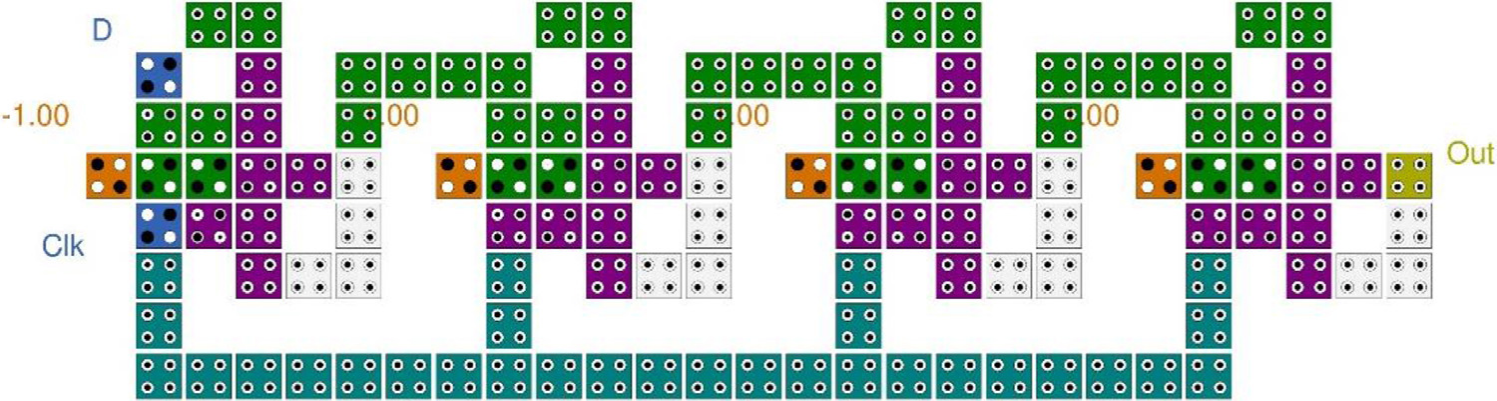
QCA based shift register using D-FF.

**Fig. 6:**
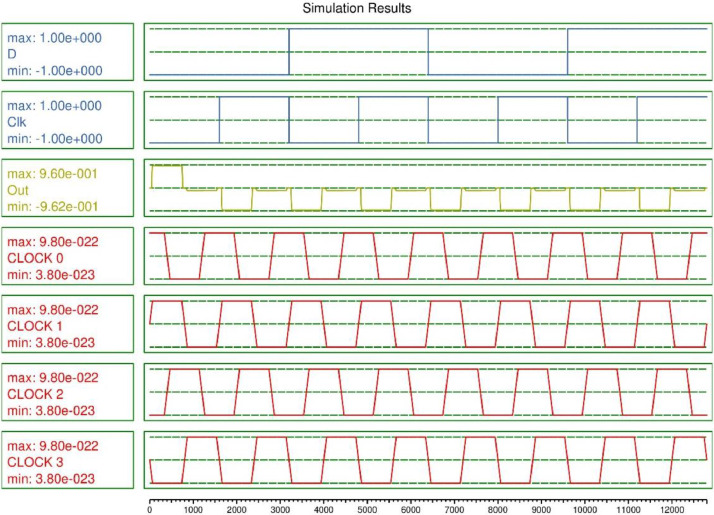
Simulation outcome of SISO register using D-FF.

In this paper, D-FF [5] and JK-FF [6] has been simulated and functionality has been validated, these are the building block of the complex sequential circuit. The 4-bit SISO register is designed using JK-FF and D-FF. The majority gate based flip flops are demonstrated, the QCA layout is designed in the software and simulation outcomes are illustrated in Fig. 1, Fig. 2. The D-FF and JK-FF based SISO circuits layout and simulation outcomes were examined from Fig. 3, Fig 4, Fig. 5, Fig. 6. The structural analysis of the flip flops was carried out in Table 1. The comparisons of various SISO structure were demonstrated in Table 2. The Quantum cost of the QCA based registers designs were compared.

**Table 1:** Comparison of Flip Flops.

FF	Complexity	Area (nm)	Total Area (nm^2^)	Ref	Complexity	Area(nm)	Total Area (µm^2^)	Latency	Quantum Cost (Latency)2x Area
JK	50 [Ref 2]	198×318	62,964	[5]	39	234×178	0.04	0.5	0.01
D	48 [Ref 3]	218×218	47,524	[6]	20	195×121	0.02	1	0.02

**Table 2:** Design analysis of Serial in Serial Out.

Proposed design	Complexity	Area	Latency	Cost
Shift register using D-FF	122	0.10 µm^2^	1	12.2
Shift register using JK-FF	216	0.19 µm^2^	1	41.04

## 3 Experimental Design, Materials and Methods

The default parameter of QCA Designer tool ver.2.0.3 is employed to verify the functionality of the QCA based logic circuits. The Quantum Dot diameter was set at 5 nm with a radius of effect of the cell is 65 nm and the number of samples 12,800 with convergence Tolerance 0.001000. The bistable approximation Engine [4] is implemented to simulate the circuits in the QCA Designer tool [1] the maximum iteration per sample is 100.

The simulation outcome explained in Fig. 1(c) confirms the operational functionality of the QCA based JK-FF is the main functional block of shift register. The output produces is expressed as:

(1) $$\text{JQ}{}^{\prime }+K{}^{\prime }Q$$

The latency of the circuit is 0.5 which is 78% less than the previous design [2].

20 QCA cells were utilized to design the D-FF with an area occupancy 0.02 µm^2^ with latency 1. It occupies 42% less area than the previous design [3]. The simulation outcome is illustrated in Fig. 2(c). The waveform demonstrates the functionality of the D flip flop with the delay of 1 clock cycle. The D-FF and JK-FF are the best candidates to design the 4-bit SISO.

The layout of JK-FF based 4 bit shift register is demonstrated in Fig. 3. The validated functionality of the layout is illustrated is explained in Fig. 4. The QCA based SISO is designed using 4 JK-FF occupies 238 QCA cells and utilizing 0.20 µm^2^. The D-FF based 4 bit shift register is constructed with 122 QCA cell and utilizing 0.10 µm^2^ as demonstrated in Fig. 5 and functionality is validated in the Fig. 6. The quantum cost is important aspect while designing the QCA based circuits which is calculated as cost = (Latency)2 × Area [7]. The structural analysis on the basis of Area, Complexity, and cost is demonstrated in Table 1 and compared with the previously published work.

The performance comparison of D-FF and JK-FF is carried out in Table 1 which indicates that proposed structures are having lower complexity and lower quantum cost for the design of complex sequential QCA-based circuits. Hence the shift register constructed using these flip flops indeed had lower complexity and lower cost.

## Limitations

Not applicable.

## Ethics Statement

The authors have read and follow the ethical requirements for publication in Data in Brief and confirming that the current work does not involve human subjects, animal experiments, or any data collected from social media platforms.

## CRediT authorship contribution statement

**Birinderjit Singh Kalyan:** Conceptualization, Methodology, Software. **Balwinder Singh:** Writing – review & editing. **Rekha Devi:** Formal analysis.

## Data Availability

Data on Quantum Dot Cellular Automata-based Flip Flops for Designing Serial-in-Serial-Out Shift Register (Original data) (Mendeley Data)
